# Endodontic Management of a Maxillary Molar with Three Mesiobuccal Canals

**DOI:** 10.1155/2014/320196

**Published:** 2014-11-19

**Authors:** Sirisha Gundam, Radhika Maddu, Sindhura Reddy Gurram

**Affiliations:** ^1^Narayana Dental College, Chinthareddypalem, Nellore, Andhra Pradesh 524003, India; ^2^SVS Institute of Dental Sciences, Appannapally, Mahabubnagar, Telangana 509002, India

## Abstract

It is imperative that the clinician should have comprehensive knowledge about the normal anatomy and its variations of the teeth as the deviations from the usual are very common. An increased awareness of unusual anatomy and a better understanding of the root canal system guide the clinician in accurate diagnosis and treatment of such variations in order to achieve a successful endodontic outcome. The maxillary first molar has been shown to have a wide variation in respect to the number of canals specifically noted in the mesiobuccal root. The current case report shows the successful management of a maxillary molar in which the mesiobuccal root had three canals.

## 1. Introduction

Knowledge of canal morphology and its frequent variations is a prerequisite for endodontic therapy. These morphological variations in root canal anatomy play a significant role in the outcome of root canal therapy. The foremost common causes of treatment failures in permanent maxillary first molars have been attributed to failure in detecting additional canals especially in the mesiobuccal root [1].

Mesiobuccal root of the maxillary first molar is studied extensively in literature. Weine et al. studied the mesiobuccal root of the maxillary first molar as the type specimen and proposed the first clinical classification of more than one canal system in a single root [1].

Studies specifically addressing the mesiobuccal root have reported that the incidence of extra root canals in vitro is greater than in vivo. Many of these in vitro studies of the mesiobuccal root canal anatomy reported the presence of a second canal but very few mentioned a third canal [2–4].

Two such studies reported their incidence to be between 1.1% and 10% [5, 6]. However, its presence has been documented in a few case reports. A case study of 140 extracted maxillary teeth has reported presence of three mesiobuccal canals in one tooth [7]. Ferguson and Favieri et al. reported maxillary molars with three mesiobuccal canals with aid of surgical operating microscope [8, 9]. Adanir also reported a similar case with four roots and six canals [10]. Martinez-Berna and Ruiz-Badanelli and Beatty reported the maxillary first molar with three separate mesiobuccal canals with separate foramina [11, 12]. Kottoor et al. reported two maxillary first molars with three mesiobuccal canals in each tooth with the aid of Cone Beam Computed Tomography (CBCT) [13, 14]. These case reports have been summarised in the table below (Table 1). The documentation of these case reports may facilitate the recognition and successful management of similar cases requiring endodontic therapy.

Based on statistical analysis, an association exists between ethnicity and number of roots and root canals in Caucasian, Indian, Mongoloid, and Middle Eastern population groups [15]. In second premolars, Caucasian, Indian, and Middle Eastern populations showed a higher prevalence of multiple canals (14–17%) [16].

The present study reports the successful management of an unusual maxillary first molar with five canals in which the mesiobuccal root has three canals.

## 2. Case Report

A 19-year-old girl of Indian descent presented to Department of Endodontics and Conservative Dentistry with pain, which was continuous and severe on intake of hot foods since 3 days. The patient's medical history was noncontributory. Clinically the right maxillary first molar had a deep carious lesion. Electric pulp testing suggested irreversible pulp damage.

After clinical and radiographic examination, nonsurgical endodontic therapy was initiated. The patient received local anesthesia of 2% lidocaine with 1 : 100,000 epinephrine. After rubber dam isolation, a conventional endodontic access opening was made. Clinical evaluation of the pulp chamber revealed 3 principal root canal systems: mesiobuccal (MB), distobuccal (DB), and palatal (P). After probing with a Hu-Friedy (Chicago, IL) DG 16 endodontic explorer, a small hemorrhagic point was noted in a groove approximately 2 mm from the MB orifice in a palatal direction. A similar hemorrhagic point was noted at 2 mm further palatally from the second MB canal. A small amount of dentin that was occluding the orifice of the third mesiobuccal was removed.

**Figure 1:**
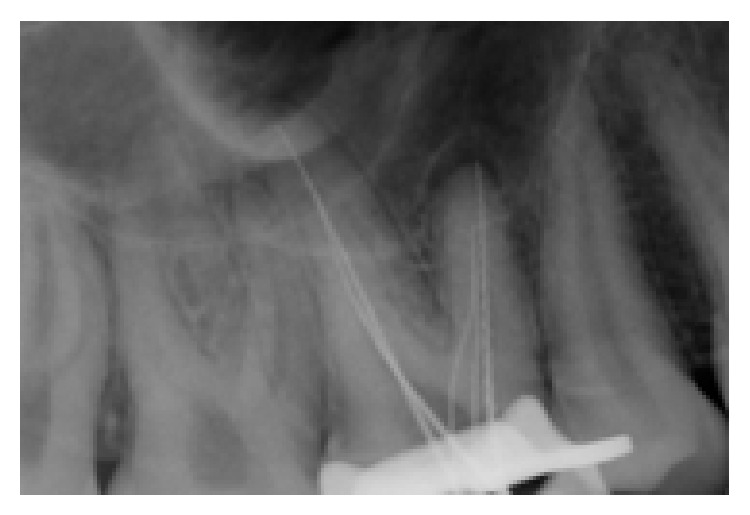
Working length radiograph showing three MB canals. MB3 joining the MB2 at middle third.

**Figure 2:**
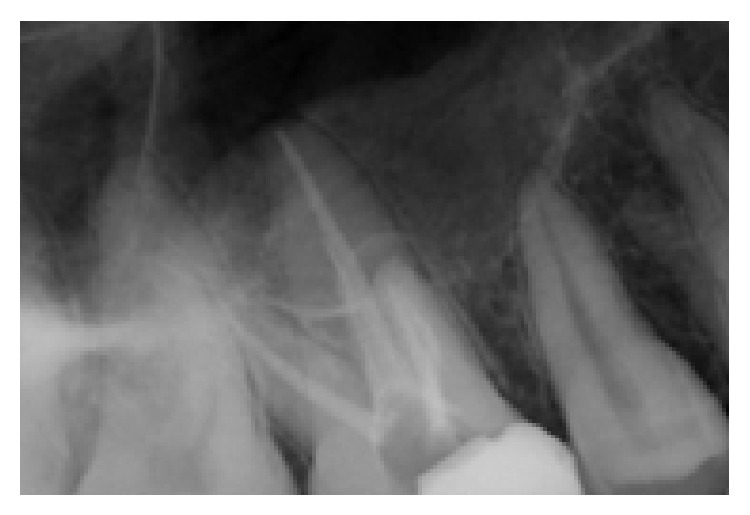
Postobturation radiograph showing all 3 MB canals exiting as single canal.

The conventional triangular access was modified to a trapezoidal shape to facilitate access to these additional canals. The working lengths of all five canals were estimated by means of an electronic apex locator (Dentaport ZX Morita, Tokyo, Japan) and then confirmed by a digital radiograph. The third mesiobuccal canal joined the second mesiobuccal canal at the middle third and continued as single canal. Digital radiovisiograph (Kodak RVG system) at 20 degree angulation revealed that MB1 and MB2 canals merged in the apical third (Figure 1). The canals were initially instrumented with #15 stainless steel *k* files (Mani Inc, Tochigi, Japan) under irrigation with 5% sodium hypochlorite (Prime Dental Products, Thane). All canals were cleaned and prepared by Protaper rotary nickel-titanium files with a Crown-down technique (Dentsply Malliefer). Calcium hydroxide (Prime Dental Products, Thane) was placed as intracanal medicament and access cavity was sealed temporarily with IRM (Caulk Dentsply, Milford, USA). One week later, the tooth was asymptomatic; obturation was done with Endomethasone sealer (Septodont, US) and Guttapercha (Dentsply Malliefer). A postobturation radiograph was obtained (Figure 2).

## 3. Discussion

The most common cause of root canal treatment failures in permanent maxillary first molars has been attributed to failure in locating additional canals especially in the mesiobuccal root and therefore has resulted in more research and clinical investigation than any other tooth [17].

It presents with wide variations in its anatomy with respect to frequency of occurrence of the number of canals in each root and the number of roots. The failures in root canal therapy of the permanent maxillary first molars are mainly due to the difficulty in locating and filling the second and/or third mesiobuccal canals [18, 19].

The mesiobuccal root of the maxillary first molar is broad mesiopalatally unlike the distobuccal root which is round in cross-section. This anatomic difference could possibly explain for the higher incidence of multiple canals in the mesiobuccal root [20]. The incidence of two mesiobuccal canals in maxillary molar ranges from 53 to 95% but the presence of third canal has been barely reported in the literature [8].

Diagnostic measures such as multiple angled preoperative radiographs, examination of the pulp floor with a sharp explorer, troughing of grooves with ultrasonic tips, visualising the haemorraghic points, staining the chamber floor with 1% methylene blue dye, and hypochlorite champagne bubble test are few important aids in locating root orifices.

In the present case, examination of the pulpal floor and exploration of haemorrhagic points with the DG16 hinted at presence of extra orifices and canals. A hemorrhagic point was noted at 2 mm palatally from the second MB canal. The third mesiobuccal canal joined the second mesiobuccal canal at the middle third and continued as single canal. Digital radiovisiograph at 20 degree angulation revealed that MB1 and MB2 canals merged in the apical third unlike the case reported by kottor, wherein the mesiobuccal root showed a Sert and Bayirli type XV canal configuration.

Radiographic examination is the most vital constituent in the management of endodontic problems. Images taken in 20 degrees angulation from mesial and distal side reveal the basic information on the tooth's anatomy and variations in root canal system. They provide a clue to the type of canal configuration in spite of its inherent limitations. Newer diagnostic methods such as CBCT scanning greatly facilitate access to the internal root canal morphology. Matherne et al. investigated the use of CBCT and concluded that CBCT images always resulted in the identification of greater number of root canal systems than digital images. Although conventional CT scans produce a high level of detail in the axial plane, it is essential that the radiation dose is kept as low as reasonably achievable [21].

Operator experience has a significant role in locating and negotiating difficult canals in the MB root of maxillary molars in favor of experienced operator [22]. Operator should take more time during the appointment to search for additional canals. Clinically, if the files are off centered during the exploration or in the working length radiograph, operator can be suspicious of presence of additional canals in the root [23]. In this case, the third canal was located by modifying the access cavity from the traditional triangular outline form to a rhomboidal shape which permitted straight line visualization, allowing for complete debridement of the pulp chamber and aided in localization of the MB2 and MB3 canal in the mesiobuccal root of the maxillary first molar. The young age of the patient along with the modified access preparation in this case could be the reason for the relative ease of identification of the third canal without the use of a surgical operating microscope. Thorough knowledge of complexity of the root canal system and its variations, increased operator experience, and increased time per appointment with adequate illumination help in identification and treatment of these extra canals.

Studies with modern techniques supported by Dental Operating Microscope and adequate illumination have reported higher rates of detection of a second/third canal in the mesiobuccal root of maxillary molars [4]. If thorough exploration of the tooth is forgone, the presence of these extra canals could be potentially missed leading to treatment failure.

Advanced imaging technologies like spiral computed tomography (SCT) and cone-beam computed tomography were used in doubtful circumstances as an adjunctive aid for detection and management of the variations in root canal morphology [22]. In the present case, radiographs of different angulations and clinical examination of the floor of the pulp chamber clearly depicted the variable anatomy. Hence, advanced imaging techniques (SCT and CBCT) were not used. In spite of providing an excellent insight into the anatomical variations of the root or root canal configuration, these imaging modalities also potentially increase the effective dose of radiation exposure for the patient.

The clinician should have thorough knowledge of the root canal morphology and its abberations. The maxillary molar presents a wide variation in respect to the number of canals. The prognosis of first molars depends on detection of the extra canal(s) and their proper cleaning, shaping, and sealing. Failure to detect and treat a canal might cause treatment failure.

## 4. Conclusion

Thorough knowledge of complexity of the root canal system and its variations increased operator experience and increased time per appointment with adequate illumination help in identification and treatment of these extra canals.

## Figures and Tables

**Table 1:** 

Author reference	Number of roots	MB	DB	P	Number of canals	Ethnicity/age
Martinez-Berna and Ruiz-Badanelli (1983) [11]	3	3	2	1	6	Spanish, 10 and 17 yr old male
Beatty (1984) [12]	3	3	1	1	5	US, 14 yr old male
Ferguson et al. (2005) [8]	3	3	1	1	5	US, 18 yr old male
Favieri et al. (2006) [9]	3	3	1	1	5	Brazil, 15 yr old male
Kottoor et al.(2010) [13]	3	3	2	2	7	Indian, 37 yr old male
Kottoor et al.(2011) [14]	3	3	3	2	8	Indian, 30 yr old male
